# Incidence of proximal tibia fractures in adults in Sweden show higher rates in women and a marked increase among young women

**DOI:** 10.1038/s41598-026-39751-6

**Published:** 2026-02-12

**Authors:** Fredrik Olerud, Anne Garland, Nils P. Hailer, Olof Wolf

**Affiliations:** Department of Surgical Sciences, Orthopaedics & Hand Surgery, Uppsala University, Uppsala, Sweden

**Keywords:** Diseases, Health care, Medical research

## Abstract

Proximal tibia fractures (PTF) account for ~ 1% of all adult fractures and show a bimodal age distribution, occurring after high-energy trauma in younger individuals and low-energy fragility fractures in the elderly. Contemporary nationwide data on incidence and management are lacking for Sweden. The aim of the present study was therefore to investigate the incidence and management of PTFs in Sweden using nationwide register data. Specifically, we sought to (1) determine the incidence of PTFs over time, (2) describe demographic characteristics of affected patients, and (3) analyse treatment patterns, including the use of acute arthroplasty. Using the Swedish National Patient Register, all patients *≥* 18 years of age, with a diagnosis of PTF (ICD-10 S82.1) between 2011 and 2023 were included. Incidence rates were calculated using national population data and reported per 100,000 person-years. Patient demographics and treatment patterns, including surgical methods, were analysed and compared between sex and age groups. A total of 38,053 patients with PTF were identified (60% women, mean age 57 years). The overall incidence increased from 36.7 per 100,000 person-years in 2011 to 40.1 in 2023 (+ 9.3%). Incidence remained stable in men (31.0 to 30.3), while it increased by 18.5% in women (42.2 to 50.0). The highest incidence-rate throughout the study period was observed among women aged ≥ 80 years, although rates in this group declined from 111.0 per 100,000 person-years 2011 to 90.3 in 2023 (–18%). The largest relative increase occurred in women aged 20–29 years (12.7 to 25.4; +100%). Between 2011 and 2023, 29% of patients underwent surgery, most commonly plate fixation (71.6%). The incidence of PTFs in Sweden has increased modestly over the past decade, driven primarily by a rise among young women. Up to the age of 50, the majority of PTFs were sustained by men (*n* = 7849, 57.6%), whereas above 50 the vast majority were sustained by women (*n* = 17249, 70.6%). Surgical treatment rate has been stable over the 13 years study period, with the large majority being treated with plate fixation.

**Supplementary Information:**

The online version contains supplementary material available at 10.1038/s41598-026-39751-6.

## Introduction

Proximal tibia fractures (PTFs) account for approximately 1% of all adult fractures as reported in a study that examined 5,953 adult fracture cases treated at the Royal Infirmary of Edinburgh, Scotland, during the year 2000^1^. The authors reported an incidence of PTFs of 13.3 per 100,000 person-years. Although PTFs comprise only about 1% of all adult fractures, they represent approximately 8% of fractures in the elderly population^2,3^, supporting their classification as a fragility fracture. However, while research has shown that fractures of the lower leg are associated with low bone mineral density in women, a consistent age-related increase among men has not been observed^4^. PTFs have a bimodal age distribution^1^; in younger individuals they are often the result of high-energy trauma, such as road traffic accidents, whereas in the elderly population they typically occur following low-energy falls in the context of decreased bone quality.

With an ageing population, an increase in the incidence of fragility fractures in general, including PTFs, would be expected, a trend partly reflected by a reported proportion for PTFs of 2.7% of all fractures in 2022^5^as compared to 1.2% in 2000^1^. A single-center report showed a 68% increase over the decade 2011–2020 in PTFs with the highest incidence in older women^6^.

Contemporary epidemiological data on PTFs remain limited. Most available studies are regional^1,5,7^, and few have reported nationwide, population-based incidence rates and trends^8,9^. Moreover, while PTFs are recognized as a heterogeneous injury group affecting both younger and older patients, the extent to which demographic changes such as an ageing population have influenced their incidence and treatment patterns are still not fully understood.

In European populations, the majority of PTFs are managed non-surgically, while approximately 37–52% require surgical intervention^8,10,11^. The current gold standard for the treatment of displaced PTFs is open reduction and internal fixation (ORIF) using plates and screws^12,13^. Primary total knee arthroplasty (TKA) for selected, severely displaced intraarticular fractures in the elderly remain debated. Intended to enable early mobilization and full weight-bearing, it is supported by limited but promising small, single-centre series^14,15^.

The aim of the present study was therefore to investigate the incidence and management of PTFs in Sweden using nationwide register data. Specifically, we sought to (1) determine the incidence of PTFs over time, (2) describe demographic characteristics of affected patients, and (3) analyse treatment patterns, including the use of acute total knee arthroplasty (TKA).

## Patients and methods

In this observational register-based study the cohort was obtained from the Swedish National Patient Register (NPR). Data on all adult *≥* 18 patients with a recorded inpatient or outpatient visit carrying a diagnosis of PTF, International Classification of Disease − 10 (ICD-10) code S82.1 (including all subcodes), from 2010 to 2023 were extracted from the register. Although full ICD-10 subcodes (fourth character) are available in the NPR, these denote fracture openness (open vs. closed) rather than anatomical subtype. Consequently, the register does not permit reliable stratification between intra-articular and extra-articular PTFs (including tibial spine/eminence and tibial tuberosity fractures) within S82.1. The observation period was selected to provide a contemporary perspective on the epidemiology and management of these fractures. The NPR was established by the Swedish National Board of Health and Welfare in 1964 and has had nationwide coverage of inpatient care since 1987 and outpatient care since 2001. Its data have been extensively reviewed and validated^16^, and has a completeness of > 99%.

From the NPR, we obtained data on sex, age at the time of injury, index date (defined as the date when the diagnosis was first registered), diagnosis codes, and procedure codes. The procedure codes included, but were not limited to, external fixation (NGJ29), fixation with biodegradable implant (NGJ39), wire/pin fixation (NGJ49), intramedullary nailing (NGJ59), plate fixation (NGJ69), screw fixation (NGJ79), fixation with combined methods (NGJ89), other fracture surgery (NGJ99), and primary TKA (NGB19–99). When patients had multiple visits or hospitalizations, only the first event was included, as it was not possible to reliably distinguish new injuries from subsequent episodes related to the index injury. Patients who sustained their fracture in 2009 could appear with follow-up visits in 2010, thereby receiving an incorrect index date and artificially inflating the incidence for that year. To address this, 2010 was excluded from all calculations and graphical presentations.

The acquired register data contained only case numbers, preventing patient identification. Consequently, medical records and pre- or postoperative radiographs were unavailable for detailed analysis.

This study adhered to the Strengthening the Reporting of Observational Studies in Epidemiology (STROBE) guidelines. (Supplemental material 1)

### Statistics

Incidence rates of PTFs and surgically treated PTFs were calculated for 2011 to 2023 using annual population data obtained from Statistics Sweden (SCB), which maintains the national electronic population register. Rates are reported per 100,000 person-years and reflect the adult population of Sweden. Incidence rates were calculated for men and women, and for the age groups 10–19, 20–29, 30–39, 40–49, 50–59, 60–69, 70–79 and 80+, as well as for the entire cohort. Because the cohort was restricted to adults (≥ 18 years), crude incidence rates for the total population and by sex were calculated using adult-only denominators (total population minus individuals aged 0–17 years) obtained from Statistics Sweden. For age-stratified analyses, SCB population denominators were available only in 10-year age bands (10–19, 20–29, etc.). Consequently, the 10–19 age stratum represents a truncated case definition (18–19 years in the numerator) while the denominator reflects the full 10–19-year population, which may underestimate incidence in that stratum. This approach was applied consistently across all years and therefore does not affect within-stratum temporal trend interpretation.

In addition to crude incidence rates, age-standardized incidence rates were calculated using the direct standardization method with the 2013 European Standard Population as reference^17^. Age-standardized rates are reported per 100,000 person-years for men, women, and the total population. Because the cohort was restricted to adults, standardization was performed for ages 20 years and older (20–29 to 80+), and ESP2013 weights were renormalized across the included age strata.

As these figures represent complete population data rather than estimates derived from a cohort or sample, 95% confidence intervals and additional statistical estimation procedures were not applied. All analyses were conducted using R software (version 4.3.1).

## Results

**Fig. 1:**
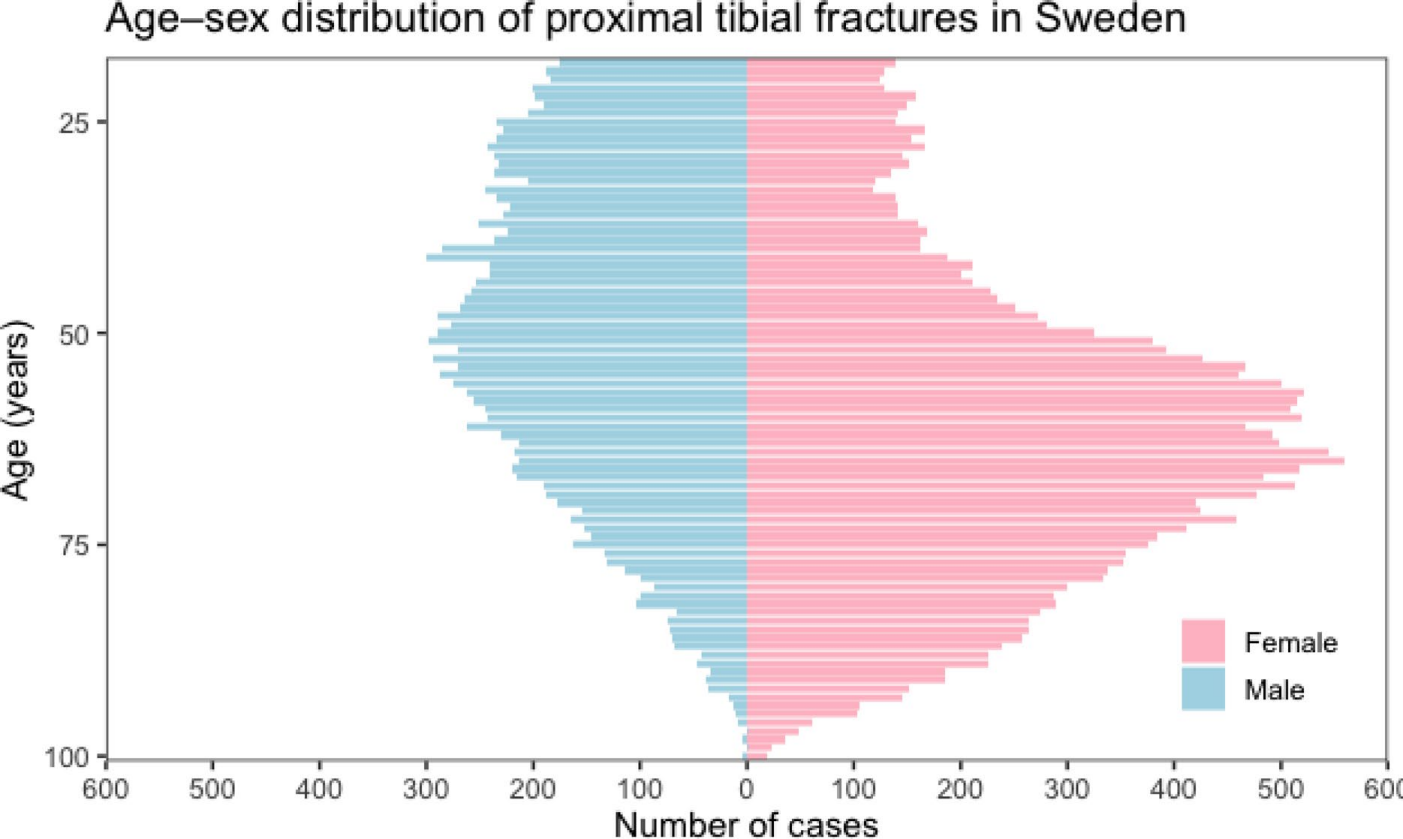
Age distribution of proximal tibia fractures population in Sweden 2011–2023 (*n* = 38053).

A total of 38,053 patients (23,029 women; 60.5%) with PTF were identified in the NPR between 2011 and 2023. The mean age at injury was 56.8 years; 49.8 years for men and 61.3 years for women (Fig. 1). Mean age remained stable during the study period (men: 50.1 years in 2011 vs. 49.0 years in 2023; women: 62.8 vs. 59.9 years). Among surgically treated patients, the mean age was 49.5 years in men and 60.0 years in women, with little variation over time (men: 49.6 vs. 50.2 years; women: 60.3 vs. 59.6 years in 2011 and 2023, respectively).

### Fracture incidence

In 2017, a total of 124,772 fractures were reported to the NPR in Sweden, of which 2,967 were classified as PTFs (2.38%). In comparison, a total of 136,461 fractures were reported in 2023, with 3,360 classified as PTFs, corresponding to 2.46% of all fractures. Data on the total number of fractures for the years 2011–2016 were not available in our dataset; consequently, trends in the proportion of PTFs over the full study period could not be evaluated.

**Fig. 2:**
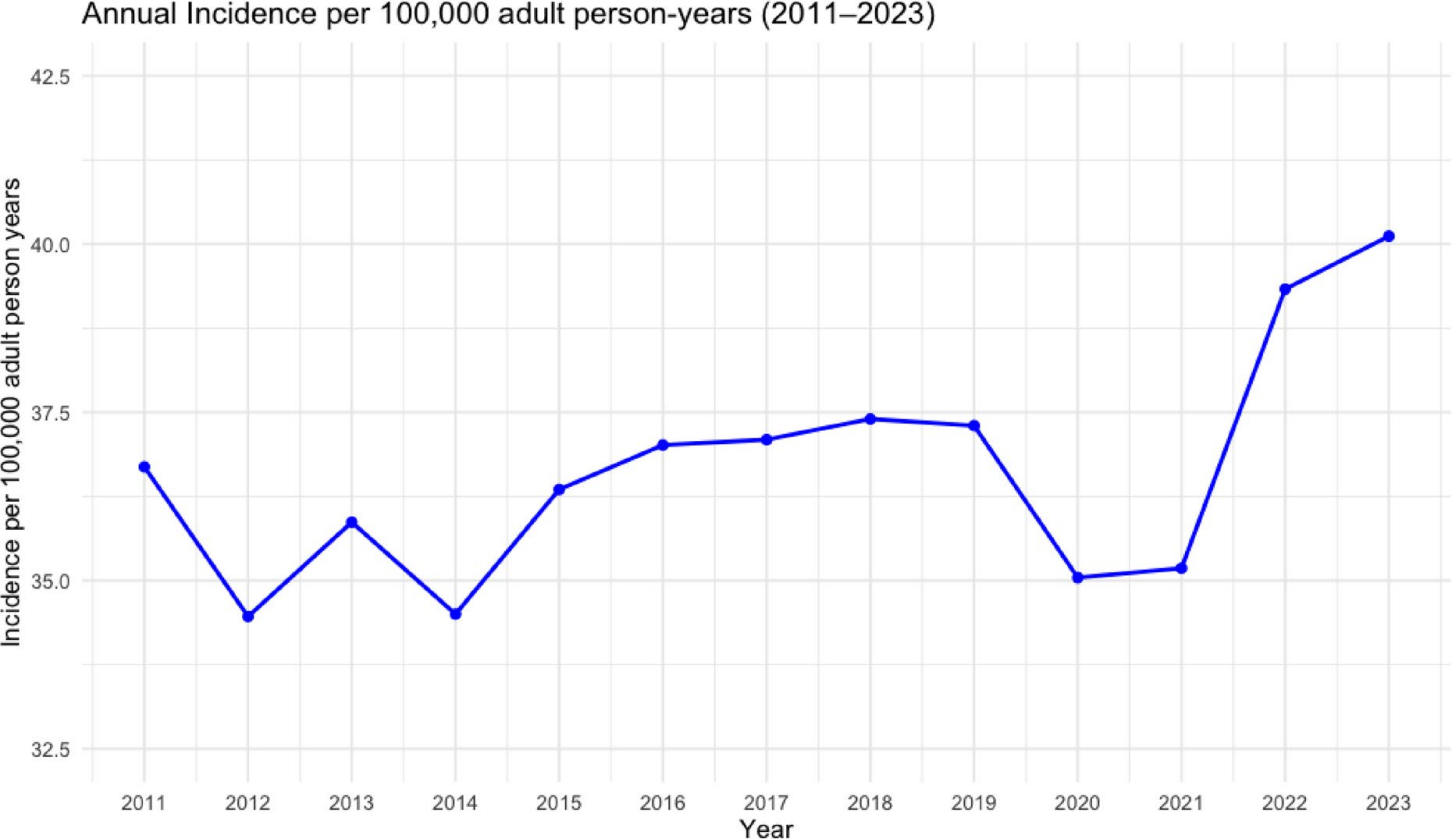
Incidence of proximal tibia fractures in Sweden 2011–2023, per 100,000 person-years.

The total incidence of PTFs in Sweden was 36.7 per 100,000 person-years in 2011 and 40.1 in 2023, corresponding to a 9.3% increase over the observation period (Fig. 2).

**Fig. 3:**
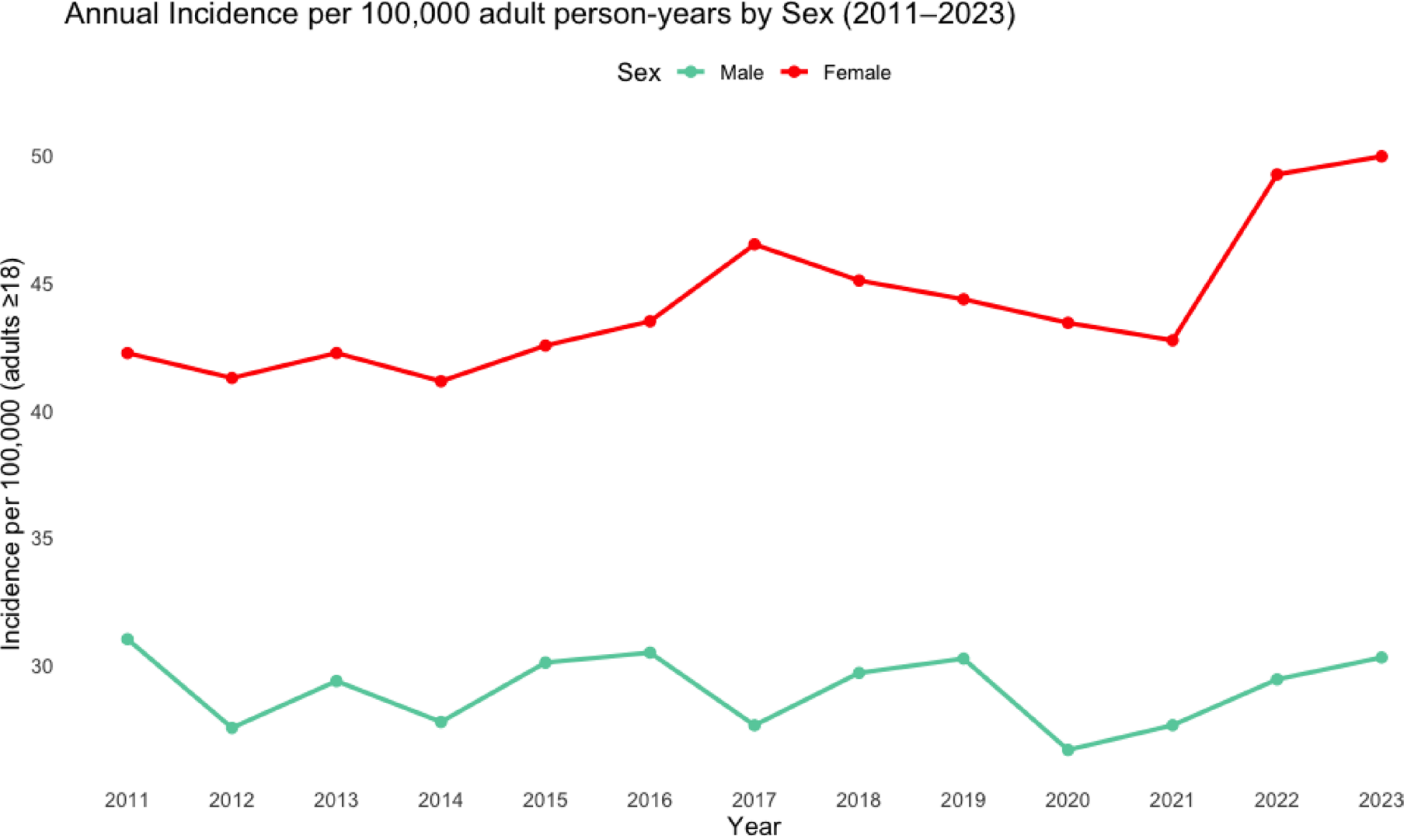
Incidence of proximal tibia fractures for men and women in Sweden between 2011 and 2023, per 100,000 person-years.

The incidence of PTFs in men was 31.0 per 100,000 person-years in 2011 and 30.3 per 100,000 person-years in 2023. In women the incidence was 42.2 vs. 50.0 per 100,000 person-years in 2011 and 2023, respectively. Thus, the incidence in men remained essentially unchanged, while the incidence in women increased by 18.5% over the study period (Fig. 3).

**Fig. 4:**
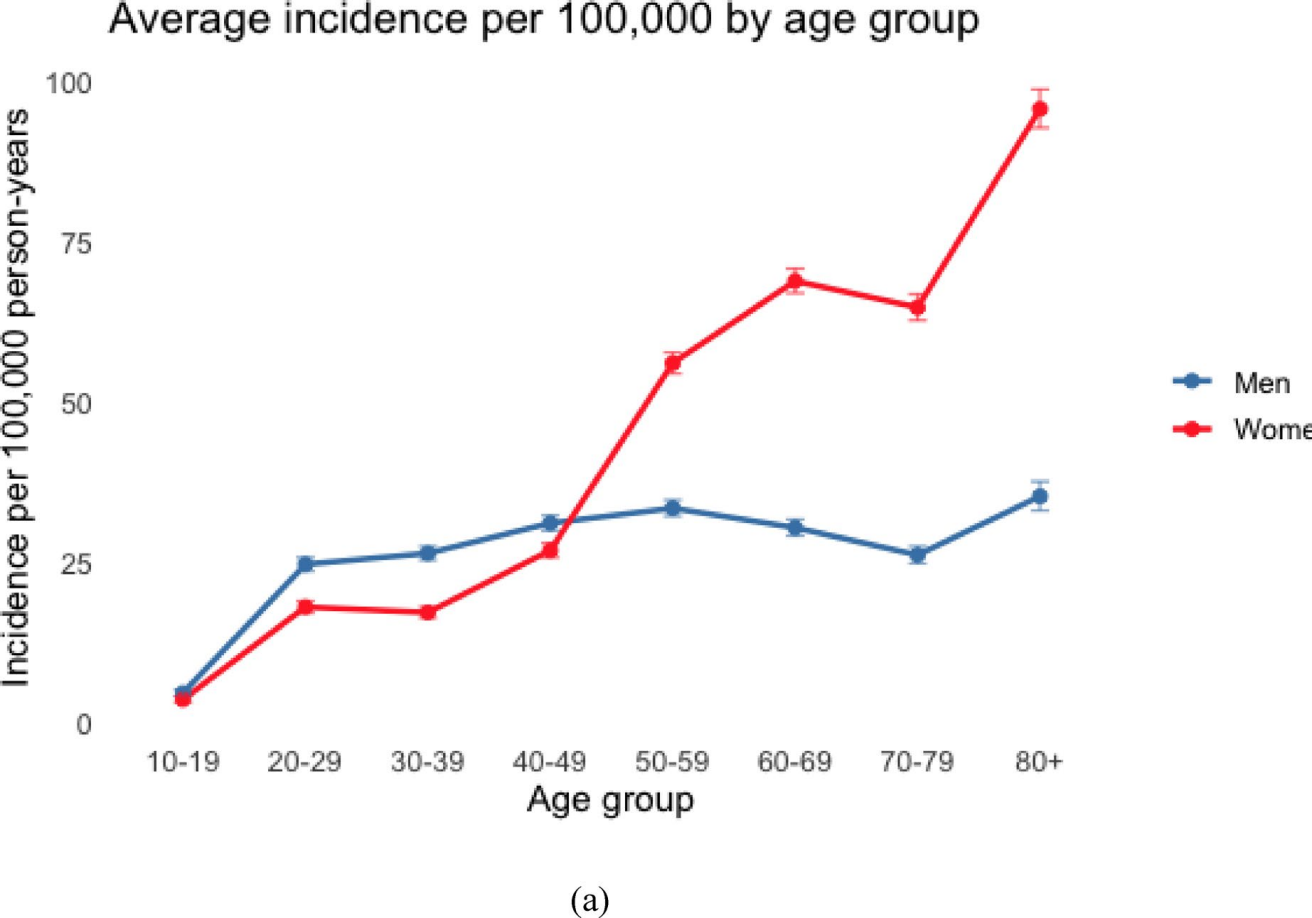

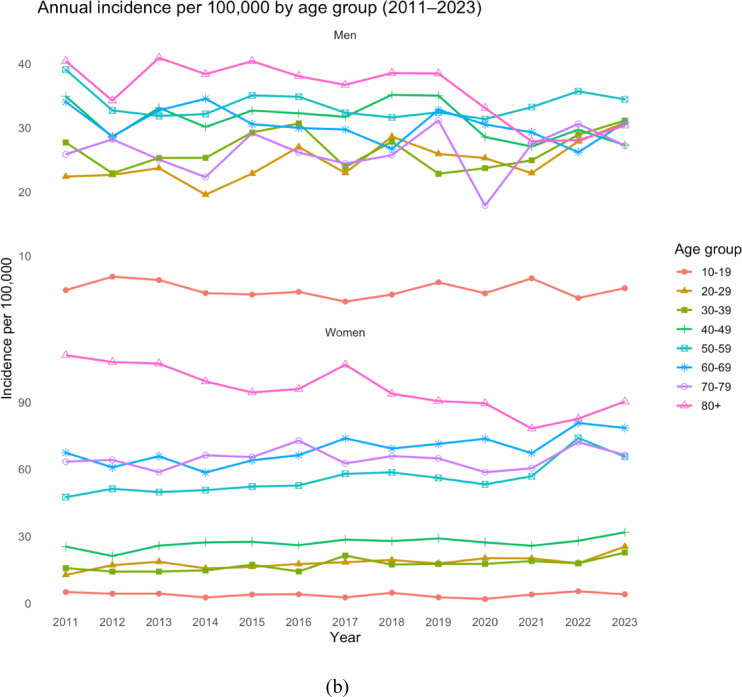
Incidence of proximal tibia fractures for men and women in different age groups in Sweden between 2011 and 2023, per 100,000 person-years. Average incidence during the entire observation period (**a**) and temporal trends (**b**).

The highest incidence was observed in women aged 80 years and older, with 111.0 per 100,000 person-years in 2011 and 90.3 in 2023. This corresponds to an 18% decrease over the study period for women ≥ 80 years. The greatest relative increase occurred in women aged 20–29 years, in whom the incidence doubled from 12.7 per 100,000 person-years in 2011 to 25.4 per 100,000 person-years in 2023 (Fig. 4).

Up to the age of 50, the majority of PTFs were sustained by men (*n* = 7849, 57.6%), whereas above 50 the vast majority were sustained by women (*n* = 17249, 70.6%).

Age-standardized incidence rates for adults aged 20 years and older demonstrated a gradual increase over the study period. The overall age-standardized incidence increased from 37.4 per 100,000 person-years in 2011 to 40.4 in 2023, corresponding to a relative increase of 8.0%. Among women, age-standardized incidence increased from 41.5 to 49.6 per 100,000 person-years, whereas rates among men remained comparatively stable (31.6 vs. 30.4 per 100,000 person-years). A transient decline was observed in 2020–2021, consistent with reduced fracture incidence during the COVID-19 pandemic (Table 1).

**Table 1:** Crude and Age-Standardized incidence of proximal tibial fractures (Adults) crude incidence (adults ≥ 18 years) and age-standardized incidence (ESP2013, ages ≥ 20 years) per 100,000 person-years in Sweden, 2011–2023. Percent change is calculated relative to 2011.

Year	Crude men	Crude women	Crude total	Crude % change vs. 2011	ASR20 men	ASR20 women	ASR20 total	ASR20% change vs. 2011
2011	31.0	42.2	36.7	0.0%	31.6	41.5	37.4	0.0%
2012	27.5	41.3	34.5	−6.0%	27.7	40.8	35.0	−6.4%
2013	29.4	42.2	35.9	−2.2%	29.6	41.6	36.3	−3.0%
2014	27.8	41.1	34.5	−6.0%	28.3	41.0	35.2	−5.9%
2015	30.1	42.5	36.4	−0.8%	30.8	42.2	37.0	−1.1%
2016	30.5	43.5	37.0	+ 0.8%	31.0	43.0	37.5	+ 0.3%
2017	27.6	46.5	37.1	+ 1.1%	28.3	46.3	37.9	+ 1.3%
2018	29.7	45.1	37.4	+ 1.9%	30.2	44.6	37.9	+ 1.3%
2019	30.2	44.4	37.3	+ 1.6%	30.5	44.2	37.8	+ 1.1%
2020	26.7	43.4	35.0	−4.6%	27.0	43.3	35.6	−4.8%
2021	27.6	42.7	35.2	−4.1%	27.5	42.4	35.3	−5.6%
2022	29.4	49.3	39.3	+ 7.1%	29.8	48.9	39.7	+ 6.1%
2023	30.3	50.0	40.1	+ 9.3%	30.4	49.6	40.4	+ 8.0%

Percent change calculated relative to 2011 values. ASR20 = Age-standardized incidence using the 2013 European Standard Population (direct method), ages ≥ 20 years.

Crude incidence calculated using adult population denominators (≥ 18 years).

### Treatment

**Fig. 5:**
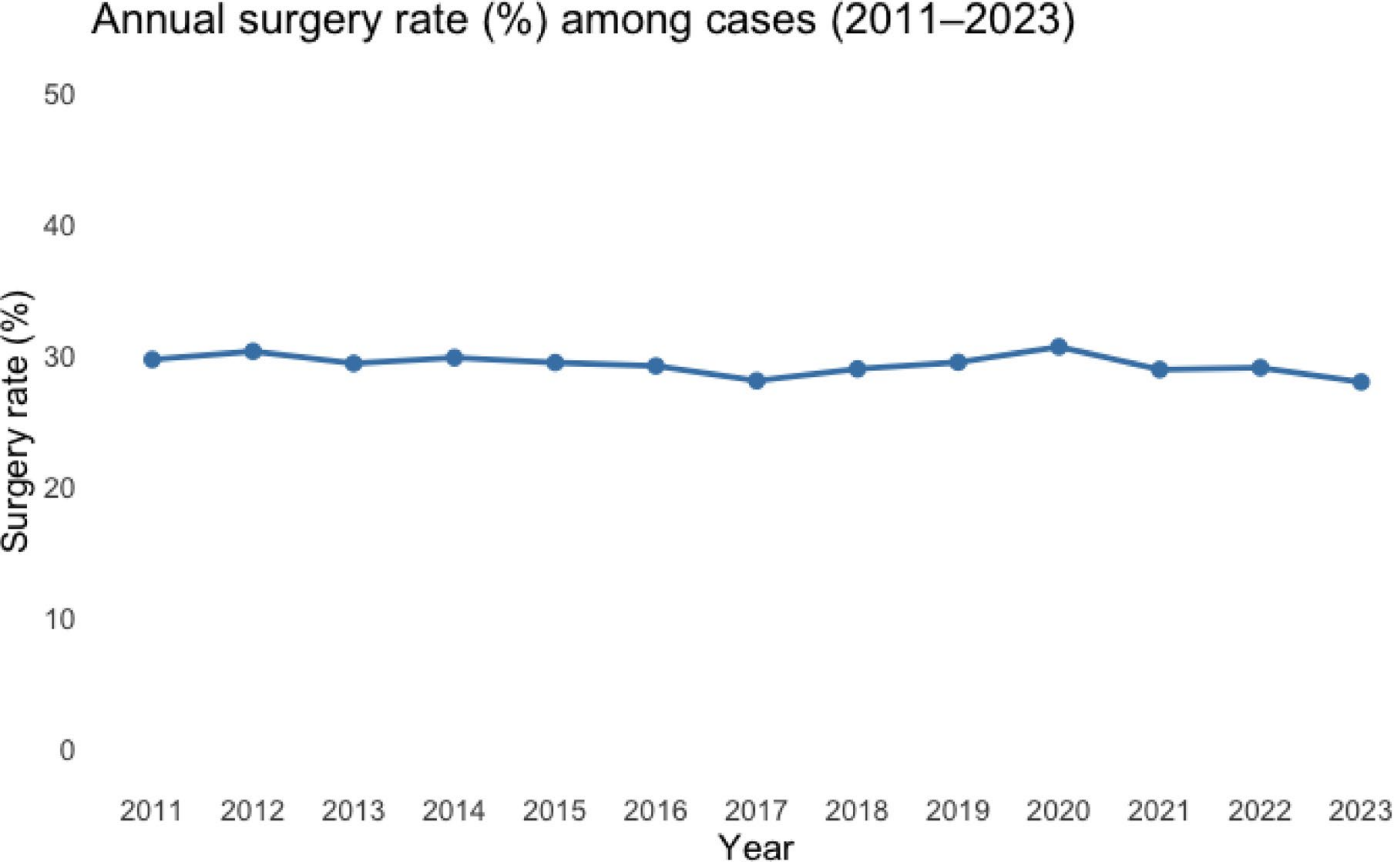
Surgery rate for proximal tibia fractures in Sweden 2011–2023.

Between 2011 and 2023, 11,141 patients (29.3%) were treated surgically, of whom 6,716 (60.3%) were women. The proportion of surgically treated fractures remained stable over time, with 29.7% in 2011 and 28.0% in 2023 (Fig. 5). Over the whole study period, ORIF with plate and screw fixation was the most used method (*n* = 8,083; 72.5%), followed by intramedullary nailing (*n* = 736; 6.6%), fixation with screws only (*n* = 726; 6.5%) and primary arthroplasty (*n* = 467; 4.2%).

**Fig. 6:**
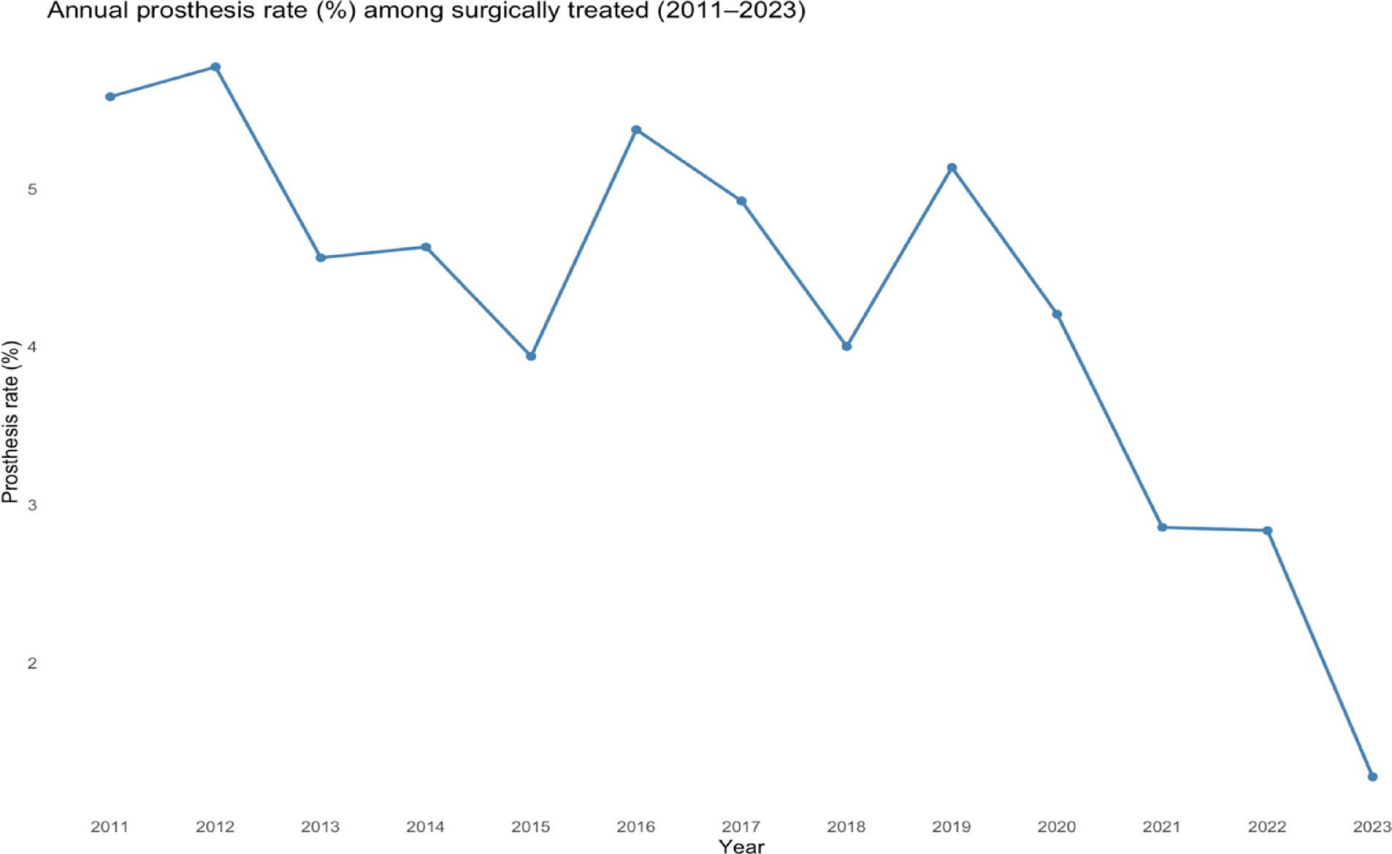
Frequency of proximal tibia fractures treated with primary total knee arthroplasty 2011–2023. (*n* = 467).

Primary TKA as a primary treatment choice declined from 5.6% in 2011 to 1.3% in 2023 (Fig. 6).

## Discussion

This population-based observational study with data from the Swedish NPR aimed at determining the contemporary national incidence of PTFs over a 13-year period. In 2023, the measured incidence of PTFs was 40.1 per 100,000 person-years (30.3 for men and 50.0 in women), representing the highest reported incidence to date in the literature. This finding aligns with the hypothesis that, as a fragility fracture, the incidence of PTFs would be expected to rise over time in an aging population.

### Fracture incidence

Several studies report on local cohorts of patients, but national cohort studies are rare. Court-Brown et al. studied a local cohort of 5,953 patients in Edinburgh, of whom 71 sustained PTFs, corresponding to an incidence of 13.3 per 100,000 person-years and a proportion of 1.2% of all fractures in 2000^1^. Bilge et al. analysed a cohort of 5,324 patients between 2010 and 2014, identifying 157 PTFs^5^. Although incidence was not reported, the proportion had increased to 2.7%, which is closer to our measured proportion of 2.46% of all fractures in the NPR in 2023. Wennergren et al. studied 1,324 patients with tibia fractures in Gothenburg between 2011 and 2015, of whom 741 had PTFs, and reported an annual incidence of PTFs of 26.9 per 100,000 persons^7^. A national study from Belgium reported a comparable annual incidence of 29 per 100,000 person-years in 2018^8^. These figures are more in line with our observed incidence of 40.1 per 100,000 person-years in 2023 in Sweden. A recent multi-centre study from Germany examining incidence trends between 2009 and 2019 reported an average incidence of 28.7 per 100,000 person-years and an increase between 2009 and 2019 of 9%^9^.

When examining temporal changes, we observed a gradual but distinct increase in overall incidence, with a dip in 2020–2021 likely attributable to the COVID-19 pandemic, as previously reported for PTFs^18^, and also seen for ankle fractures^19^. Stratification by sex in our material revealed no increase in incidence among men. Men were younger at the time of injury, a finding that may reflect a higher general proportion of high-energy trauma in this group. In contrast, the incidence among women increased from 42.2 to 50.0 per 100,000 persons per year. This pattern supports the hypothesis that the fragility-related component of the bimodal distribution is driving the overall increase in incidence over time.

When examining age groups, we found the largest absolute increase in case counts among patients aged 50–79 years. However, the highest relative increase was observed in women aged 20–29 years, which may reflect increasing high energy trauma in this newer generation of young women, who may also be more involved in risk sports, however, as no information on injury mechanism was obtainable, the cause of this increase remains speculative.

The observed increase in crude PTF incidence reflects both demographic ageing and changes in age-specific fracture risk. Importantly, age-standardized incidence rates demonstrated a true increase in fracture risk that cannot be explained solely by population ageing. This increase was driven primarily by women, whereas age-standardized rates among men remained stable. These findings indicate that factors beyond demographic change, including behavioural or lifestyle-related risk factors, may be contributing to the rising incidence of PTFs, particularly among women.

### Treatment

The observed surgical treatment rate of 29% is lower than that reported in previous cohorts of PTFs, where approximately 40–50% have been described. Tapper et al. used the Finnish Hospital Discharge Register to assess the risk of conversion to TKA after PTF regardless of primary treatment and reported that 52% of 7,701 had initially been treated surgically^11^. In a previous report from our group, we analysed 12,012 patients with PTF in the Swedish Fracture Register (SFR) and found that 5,592 (44%) underwent surgery^10^. However, only intraarticular PTFs, i.e. AO type B and C fractures, were included in that study, whereas in the present study all PTFs, being intra- or extraarticular, share the same diagnosis code (S82.1), precluding such stratification and likely explaining part of the discrepancy. This discrepancy is likely further explained by differences in case ascertainment between registers. The Swedish National Patient Register has a completeness exceeding 99% for inpatient and outpatient care, whereas the SFR has a reported completeness of approximately 60% for PTFs^20^. Consequently, the present cohort is likely to include a broader spectrum of injury severity, including minor and non-displaced fractures that would be less likely to be captured in fracture-specific quality registers. Whether this reflects overclassification in the NPR, underreporting in fracture registers, or a combination of both cannot be determined. However, as reporting practices in the NPR are unlikely to have changed substantially during the study period, the observed temporal trends in treatment patterns remain valid. Herteleer et al. conducted a nationwide incidence analysis in Belgium (2006–2018) and reported a surgical treatment rate of 37%^8^. Given the methodological similarities, the remaining differences are most likely attributable to variation in treatment traditions between countries. Single-centre studies report higher rates of surgical intervention, Borman et al. reported a rate of 76%^6^, potentially reflecting selection bias.

We found that the proportion of surgically versus non-surgically treated PTFs remained stable over the study period. ORIF by plate and screw fixation was by far the most frequently employed surgical method, confirming its role as the gold standard in contemporary management. Treatment by intramedullary nailing, screw fixation and TKA was approximately performed at similar rates but presumably for different types of fractures. Interestingly, despite the ongoing debate regarding the potential benefits of acute TKA for displaced PTFs in elderly patients^21–24^, we observed a gradual decline in the use of primary TKA as an acute treatment strategy. This may be confounded by detection bias as it is reliant on diagnosis coding at the time of surgery, and the condition “post-traumatic osteoarthritis due to a previous fracture” may still have received the code S82.1 (PTF), regardless of time elapsed since initial fracture. Also, excluding 2010 to exclude follow-up visits from our analyses might not have been enough as some conversions to TKA after failed primary treatment may have been included as first entries in the 2011 cohort, and with a decreasing rate in subsequent years. Retrospective studies with small populations of primary TKA have reported promising results^14,15,25,26^, but randomized clinical trials and large cohort studies are still lacking. This likely contributes to hesitancy in adopting acute TKA more widely, reflecting uncertainties regarding complication risks, long-term outcomes, and the absence of high-quality evidence. Acute TKA is still an uncommon procedure in Sweden and the observed rates of acute TKA should be interpreted with caution.

### Strengths and limitations

A key strength of this study is the use of nationwide register data covering both inpatient and outpatient care, providing complete coverage of all PTFs in Sweden during the study period. Consequently, the reported incidence rates represent true population-based figures rather than regional estimates. The Swedish NPR has been thoroughly validated and shown to have high completeness and accuracy, further supporting the reliability of our findings^16^.

A clear limitation of this study is that the data extracted from the NPR did not include comorbidities such as diabetes, osteoporosis, or other chronic conditions that may influence both the risk of sustaining a fracture and the choice of treatment. Another limitation is that the current ICD-10 coding does not distinguish between different subtypes of PTFs, even though certain fracture characteristics necessitate surgical treatment to a greater extent than others. A previous study conducted by our group investigated PTFs registered during the step-wise introduction of the SFR between 2012 and 2023^10^. In that study, 15,603 PTFs were identified, compared with 35,278 cases in the present study over the same period. The SFR provides more detailed information regarding injury characteristics, fracture classification, treatment and mortality, but its completeness is lower compared with the NPR which makes incidence calculations impossible^20^. The observed discrepancy in case numbers can largely be explained by the SFR’s gradual increase in national coverage since its introduction in 2011.

The exclusion of 2010 in statistical analyses to exclude follow-up visits and later surgical procedures due to non-unions or post-traumatic symptoms may not have been enough since many conversions occur later than 1 year after primary treatment^10,27^.

The absence of laterality coding in the NPR may lead to a substantial underestimation of fragility fractures among individuals at highest risk. Because in this study each patient can only be registered once, subsequent fractures, whether refractures or those occurring in the contralateral limb, are not captured. In a previous study from the SFR on tibia fractures, including proximal, shaft and distal fractures, 2% of patients had more than one tibia fracture at the same time, and 1% sustained another tibia fracture during the study period^7^. The same limitation applies to the proportion of surgically treated patients. At high-volume hospitals, immediate operative capacity is not always available, and medically stable patients who are able to remain ambulatory with crutches may be discharged home while awaiting surgery. Such patients would not be captured as having received primary surgical treatment in the registry, which may lead to a modest underestimation of the proportion of surgically treated patients. Arthroscopic procedure codes were not available in the dataset, which may have resulted in misclassification of treatment modality and a potential underestimation of the proportion of surgically treated younger patients presenting with tibial spine (eminence) fractures.

Another limitation is the exclusion of pediatric fractures. This study was restricted to adults (≥ 18 years); therefore, fractures in individuals aged 0–17 years are not captured and the reported incidence reflects adult PTFs only.

In age-stratified analyses, population denominators were available only in 10-year age bands. As a result, the 10–19 age group includes only 18–19-year-old cases in the numerator while the denominator represents the full 10–19-year population, which may lead to underestimation of incidence in this specific age band. A key limitation of the present study is the absence of data on mortality. Information regarding vital status (alive or deceased) was not available in the dataset; therefore, mortality analyses could not be performed. Previous studies have reported a standardized mortality ratio of 1.7–2.3 following PTFs in patients aged 65 years or older, indicating a substantially elevated risk compared with the general population^28^. Although specific mortality analyses for PTF are limited, similar trends have been demonstrated for other fragility fractures, such as those of the hip, pelvis^29^, and proximal humerus^30^, where an association between skeletal fracture, age and increased mortality has been consistently observed. These findings underscore the importance of recognizing PTF not only as markers of skeletal fragility but also as potential indicators of overall frailty and increased mortality risk.

A further limitation of this study is the lack of detailed information regarding mechanism of injury. Although ICD-10 external cause codes for injury mechanism were available for a subset of patients, the structure of the register data and incomplete coding precluded reliable stratification by mechanisms such as transport-related trauma, falls, or sports injuries. Consequently, analyses of injury mechanism were not feasible. This limits the ability to draw firm conclusions regarding behavioural factors, including risk-taking behaviour, and such interpretations should therefore be considered speculative.

## Conclusion

The incidence of PTFs in Sweden has increased modestly over the past decade, driven primarily by a rise among both younger and older women. Surgical management rates have remained stable, with the vast majority of patients are treated with plate and screw fixation.

## Supplementary Information

Below is the link to the electronic supplementary material.

Supplementary Material 1

## Data Availability

Data from this study is not freely available due to the Swedish Patient Data Act and the Swedish Ethical Review Act. However, data can be extracted from the national registries with an approved ethical application due to the sensitive nature of data from national quality registers.
